# Rapid Changes in Phospho-MAP/Tau Epitopes during Neuronal Stress: Cofilin-Actin Rods Primarily Recruit Microtubule Binding Domain Epitopes

**DOI:** 10.1371/journal.pone.0020878

**Published:** 2011-06-28

**Authors:** Ineka T. Whiteman, Laurie S. Minamide, De Lian Goh, James R. Bamburg, Claire Goldsbury

**Affiliations:** 1 The Brain and Mind Research Institute, University of Sydney, Sydney, Australia; 2 Bosch Institute, School of Medical Sciences, University of Sydney, Sydney, Australia; 3 Department of Biochemistry and Molecular Biology, Colorado State University, Fort Collins, Colorado, United States of America; National Institute on Aging Intramural Research Program, United States of America

## Abstract

Abnormal mitochondrial function is a widely reported contributor to neurodegenerative disease including Alzheimer's disease (AD), however, a mechanistic link between mitochondrial dysfunction and the initiation of neuropathology remains elusive. In AD, one of the earliest hallmark pathologies is neuropil threads comprising accumulated hyperphosphorylated microtubule-associated protein (MAP) tau in neurites. Rod-like aggregates of actin and its associated protein cofilin (AC rods) also occur in AD. Using a series of antibodies - AT270, AT8, AT100, S214, AT180, 12E8, S396, S404 and S422 - raised against different phosphoepitopes on tau, we characterize the pattern of expression and re-distribution in neurites of these phosphoepitope labels during mitochondrial inhibition. Employing chick primary neuron cultures, we demonstrate that epitopes recognized by the monoclonal antibody 12E8, are the only species rapidly recruited into AC rods. These results were recapitulated with the actin depolymerizing drug Latrunculin B, which induces AC rods and a concomitant increase in the 12E8 signal measured on Western blot. This suggests that AC rods may be one way in which MAP redistribution and phosphorylation is influenced in neurons during mitochondrial stress and potentially in the early pathogenesis of AD.

## Introduction

Neuronal histopathological hallmarks of AD include neurofibrillary tangles (NFT) and neuropil threads both comprised of hyperphosphorylated microtubule-associated protein (MAP) tau. NFTs and neuropil threads assemble in cell bodies and neurites respectively. Comprising a reported >85% of end-stage cortical tau pathology, neuropil threads correlate with cognitive decline [1]–[6]. The major known function of tau, like other MAPs, is its stabilization and regulation of microtubule (MT) dynamics necessary for neurite outgrowth, morphogenesis and since tau is predominantly an axonal protein, it plays an important role in facilitating MT-dependent axonal transport (for reviews see [7], [8]). Tau can also interact with the plasma membrane and may play roles in relaying signals to the cytoskeleton from the cell surface or scaffolding signaling complexes [9]. The MT directed activity of tau is regulated by phosphorylation/dephosphorylation cycles, such that phosphorylation at specific sites detaches tau from MTs and allows MT depolymerization, while tau dephosphorylation enables it to bind and stabilize MT via its MT binding domain (MTBD) [10], [11]. In AD, tau is hyperphosphorylated, the MT network is destabilized and tau self-assembles into paired helical filaments (PHFs) that form the NFT and neuropil thread structures. Over 20 phosphorylation sites have been characterized for tau, two of these are located in the MTBD at two ‘KXGS’ amino acid motifs corresponding to residues S262 and S356 [11]–[13]. Phosphorylation of these KXGS motifs is one of the earliest markers of AD pathology, readily detectable in neuropil threads with the monoclonal antibody 12E8 that recognizes these conserved motifs in both tau as well as in other MAPs [14]. In the case of tau, phosphorylation of the MTBD sites has been shown to induce MT disassembly whereby the new unbound pool of tau is susceptible to self-assembly into PHFs [12], [15]–[17].

Neuropil threads generally precede the appearance of extensive NFTs, suggesting tau first accumulates in neurites during the development of AD pathology before the proliferation of cell body NFTs [1], [4], [18], [19]. An *in vitro* cell model for neuropil thread assembly may therefore help mimic early cellular events relevant to the disease mechanism. To this end, we recently demonstrated in primary neuronal cell culture and organotypic slice culture that mitochondrial dysfunction initiates formation of 12E8-positive neuritic inclusions that co-localize with actin depolymerizing factor (ADF)/cofilin-actin rods (AC rods) [20]. These inclusions bear some morphological resemblance to inclusions observed in AD brain which also include punctuate and rod-like linear arrays of cofilin and actin aggregates throughout the neuropil [21] (reviewed in [22]). The relevance of this model lies in an increasing body of evidence that implicates disrupted energy metabolism and mitochondrial dysfunction in AD (for reviews see [23]–[27]). Reduced mitochondrial function and increased oxidative stress are reported to occur early in disease progression, suggesting that these factors could play an integral role in initiating pathological cascades that result in the varied cytopathology of AD [23]. AC rods are independently induced in cell culture by the inhibition of mitochondrial ATP generation, thus potentially linking the formation and co-localization of AC rods and MAP inclusions to mitochondrial dysfunction [20]. Moreover, interaction of MAPs and actin in the organization of the cytoskeletal network is well-documented [28]–[35] and emerging evidence suggests that these interactions may be central to the processes involved in the initiation and development of early AD pathology [20], [22], [36], [37].

Since AD neuropathology involves hyperphosphorylation and accumulation of numerous phosphoepitopes of tau and not just those in the MTBD recognized by 12E8, the aim of this study was to investigate the phosphorylation pattern and re-distribution of several other key phospho-tau epitopes in the primary neuronal culture model. We utilized the chick model since chick neurons express five forms of tau that are homologous to human tau isoforms and exhibit conserved phosphoepitopes recognized by antibodies against human tau [38]. Using a series of commercially available phospho-tau antibodies, we measured time-dependent effects of mitochondrial dysfunction on the levels and distribution patterns of these phosphoepitopes. We found that cellular ATP reduction gave rise to rapid overall dephosphorylation at most epitopes with the exception of the 12E8 epitope that exhibited persistent phosphorylation over the 120 min treatment time. Further, 12E8 was the only epitope predominantly recruited to AC rods generated under these conditions within the time frame investigated. These results highlight the MTBD of MAP/tau as a potential early player in the initiation of neuropil threads in neurons of the AD brain.

## Methods

### Ethics Statement

Donated frontal cortex and hippocampal brain tissue from normal adult and confirmed Alzheimer's disease patients were obtained from the Alzheimer Disease Research Center, University of California, San Diego, USA and approved for use by the Institutional Review Board (formerly the Human Research Committee), Colorado State University, USA (Approval ID: JR Bamburg 2001).

Experiments involving the use of animals (cell cultures derived from chicken embryos) were approved by the Animal Ethics Committee, University of Sydney, Australia (Approval ID: CS Goldsbury K00/5-2008/3/4786).

### Antibodies and reagents

Mouse monoclonal antibodies against phosphorylated tau epitopes were as follows: AT8 (which recognizes S202/T205 in the longest isoform of human tau and S193/T196 in the longest isoform of chick tau; Pierce USA), AT100 (T212/S214 in human, T203/S205 in chick; Sigma-Aldrich), AT180 (T231/S235 in human, T222/S226 in chick; Sigma-Aldrich), AT270 (T181 in human, T155 in chick; Sigma-Aldrich), 12E8 (‘KXGS’ motifs in MTBDs of tau and other MAPs. For tau, these motifs correspond to S262/S356 in human, S253/S378 in chick; Elan USA) [14]. Other mouse monoclonal antibodies included GAPDH and β-actin (Sigma-Aldrich). Rabbit polyclonal antibodies against phosphorylated tau epitopes were S214 (S205 in chick; GenScript USA), S396 (S418 in chick; Biosource), S404 (S426 in chick; Biosource), S422 (S444 in chick; Sigma-Aldrich), total tau (Dako, USA) and against chick ADF and mammalian ADF and cofilin (1439) [39]. Anti-mouse and anti-rabbit secondary antibodies included Alexa Fluor-conjugated 488, 555, 594 and 647 (Invitrogen) for immunofluorescence and horseradish peroxidase-conjugated (Amersham) for chemiluminescence detection of immunoblots.

### Cell culture and treatments

Primary chick tectal neurons were prepared from freshly dissected chicken embryos (E7), as previously described and cultured for 7 days *in vitro* (d.i.v) on poly-D-lysine-coated 30 mm culture dishes or glass coverslips [40]. For reduction of ATP, cells were treated with 1–2 µM antimycin (AM; a mitochondrial complex III inhibitor; Sigma-Aldrich) in warm phosphate buffered saline (PBS). The actin depolymerizing drug Latrunculin B (Calbiochem 428020, CA) and actin stabilizing drug Jasplakinolide (Calbiochem 420127, CA) were used at 1 µg/ml in warm PBS. Cells left without a medium change served as controls. After treatments, cells were immediately fixed for immunostaining (cells on coverslips) or lysed, harvested and prepared for Western blot analysis (cells on tissue culture dishes).

### Immunoblotting

Primary neurons were treated in duplicate and prepared for SDS-PAGE. For equal gel loading, protein concentrations were determined by the Lowry assay (BioRad). Proteins transferred to nitrocellulose membranes were detected with an ECL Western Blotting Detection System (Amersham) on a ChemiDoc XRS (BioRad). For analysis, band densities were measured using Image J (v1.38x, National Institutes of Health freeware; http://rsb.info.nih.gov/ij), background intensity was subtracted for each individual lane and normalized to GAPDH or β-actin loading controls. For quantification, blots within the linear range of detection were selected by plotting the band density versus exposure time. An average for each treatment condition was determined with error bars representing min/max intensities (where n = 2) or SEM (where n = 4). Results are presented as a percentage of the mean control band intensities.

### Microscopy

Cells were fixed with 4% paraformaldehyde at 37°C for 35 minutes, permeabilized for 90 sec with 0.05% Triton X-100 in PBS or ice-cold 80% methanol/20% PBS (1×), blocked in 5% goat serum and immunostained using primary antibodies and fluorescent secondary antibodies described above. Epifluorescence images were obtained on a Zeiss Axioplan 2 microscope, captured with a CCD camera driven by AxioVision software. Single labeled cells/sections were used to check for bleed-through in all double-label immunofluorescence studies. Confocal images were obtained on a Zeiss LSM 510 Meta driven by LSM 510 software. All captured images were converted to Tagged Image Files for subsequent analysis and presentation.

### Nuclear accumulation analysis

For quantification of cells containing nuclear accumulation of AT100 label in control, AM- and Lat B-treated conditions, a number of randomly selected fields on each coverslip were selected and cells containing nuclear tau were counted and expressed as a percentage of the total number of counted cells. Inclusion criteria for nuclear accumulation were (i) general neuron morphology appeared normal (including intact nucleus) (ii) neuron exhibited obvious co-localization of AT100 label with DAPI nuclear label and (iii) nucleus exhibited AT100 fluorescence intensity >1.5× the intensity of nuclei of surrounding, unaffected cells, as measured using Image J. Over 200 cells were counted for each condition in duplicate experiments using independently prepared cell cultures.

### Human tissue preparation

Post-mortem time to fixation in formalin (for immunofluorescence) was 3 h. For immunofluorescence, paraffin sections (6–7 µm) were deparaffinized for 20 min in Hemo-De (Fisher Scientific), rehydrated to distilled water through decreasing concentrations of ethanol, microwaved in water for 8 min, blocked with 5% goat serum for one hour and immunostained.

### Transmission Electron Microscopy

Primary chick neurons were grown on glass coverslips, treated, washed with warm PBS and fixed for 30 min with 2.5% glutaraldehyde (Electron Microscopy Sciences, USA) in 0.1 M sodium cacodylate buffer (Sigma-Aldrich). Cells were rinsed with buffer and post-fixed for 30 min in cold 1% osmium tetroxide (Sigma-Aldrich). Cells were rinsed again and stained for 2 h with 1% uranyl acetate (Sigma-Aldrich), filtered through 0.22 µm Biofil Syringe filter in 0.05 M sodium acetate (Sigma-Aldrich). To prepare TEM grids, cells were dehydrated through increasing concentrations of ethanol (v/v) (50%, 70%, 95% and ultra-dry 100%), then infiltrated with Spurr's resin in increasing ratios to 100% ethanol (1∶1, 2∶1 and 3∶1). Cells on coverslips were embedded in 100% Spurr's resin and cured at 60°C overnight. To remove coverslips, resin blocks were placed in liquid nitrogen for ∼15 sec, transferred to warm water and carefully removed. Resin blocks were section using a diamond knife (70 nm) and placed on 200-mesh copper TEM grids that were subsequently post-stained for 10 min with uranyl acetate and lead citrate. Images were acquired with a Phillips CM 120 Biofilter TEM at 120 kV.

## Results

### Mitochondrial inhibition induces rapid dephosphorylation at all MAP/tau phosphoepitopes except for 12E8

**Figure 1:**
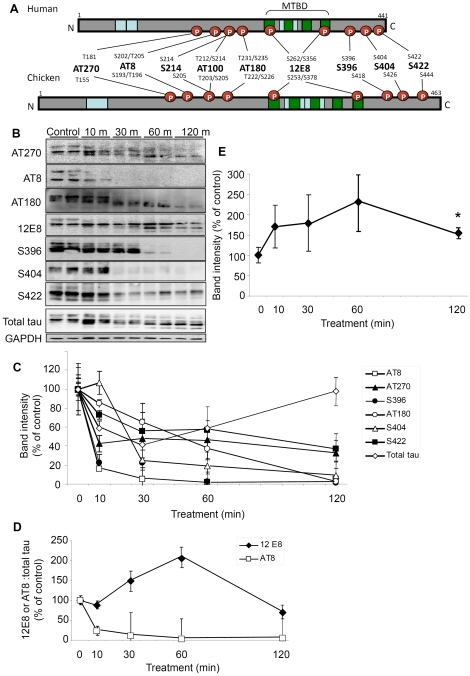
ATP reduction causes persistent phosphorylation of 12E8 epitope and dephosphorylation at other sites. (A) Epitope map of tau antibodies in human and chicken brain [38]. Schematic representations of the longest tau isoforms are shown (441 amino acid residues in human tau, 463 in chicken). The phosphorylation-dependent anti-tau antibodies (AT270, AT8, S214, AT100, AT180, 12E8, S396 S404 and S422) are shown with their corresponding target residues (S = Serine, T = Threonine). Positions of the various alternatively spliced inserts which give rise to 6 different isoforms in humans and 5 in chick are shown in blue. Green boxes denote tandem amino acid sequence repeats, which constitute the MTBD. (B) Representative Western blots of extracts from cell cultures treated in duplicate with 1 µM AM for 10, 30, 60 or 120 min. (C) Quantification of the Western blots shown in (B) reveal rapid dephosphorylation of AT8, S396 and AT270 epitopes (respectively declining to 16±0.03%, 23±9% and 43±9% of control values; mean %±min/max intensities). By 30 min, phosphorylation at epitope S404 had also markedly declined (25±11%) while epitopes S422 and AT180 exhibited a more moderate decline in phosphorylation (reduced to 55±3% and 65±20% at 30 min). Total tau was observed to decrease transiently (41±5% at 30 min) before returning to control levels (98±19%) at 120 min. All antibodies were probed on fresh blots and normalized to GAPDH loading control bands. (D) By contrast, phosphorylation of the 12E8 epitope increased markedly. To ascertain a phosphorylation∶total tau ratio, blots probed for 12E8 or AT8 (for comparison) were stripped and reprobed for total tau (polyclonal rabbit antibody). Whereas the AT8∶total tau ratio decreased rapidly, the 12E8∶total tau ratio increased at 30 min and peaked at ∼200% after 60 min. Error bars in (C and D) indicate the maximum and minimum intensities. (E) Quantification of persistent phosphorylation at the 12E8 epitope (normalized to GAPDH) was significant with a peak of >200% at 60 min and sustained phosphorylation (∼150%) at 120 min (n = 4; *p<0.05). Error bars = SEM.

In AD neurons, tau is hyperphosphorylated and abnormally redistributed to NFTs and neuropil threads in cell bodies and neurites respectively. The mechanisms by which these processes occur are not fully understood. The aim of this study was to test the hypothesis that inhibiting mitochondrial function influences the phosphorylation state and localization of tau protein. Primary neuronal cultures (prepared from E7 chick tectum) were treated for 10, 30, 60 or 120 min with the mitochondrial complex III inhibitor AM which has previously been shown to reduce the level of ATP in these neurons by 60% within 10 min [20]. Cells were then lysed or fixed for immunostaining and probed with a battery of phospho-tau antibodies - AT270, AT8, AT100, S214, AT180, 12E8, S396, S404 or S422 - that have been raised against different phosphoepitopes on human tau and have analogous corresponding epitopes in chicken tau (Fig. 1A) The 12E8 antibody recognizes phosphorylated KXGS motifs in the conserved MTBDs of both tau and other MAPs [14]. Western blot analysis of lysates revealed that compared to untreated (control) cells, ATP reduction leads to dephosphorylation of epitopes S396, AT8, AT270, AT180, S404 and S422 over the 120 min treatment time when normalized for loading to GAPDH (Fig. 1B, C). Total tau was observed to decrease transiently before returning to control levels at 120 min. By contrast, the blots revealed a strong and sustained signal for the epitopes recognized by the 12E8 antibody (Fig. 1D, E). Consistent with this, the ratio of 12E8 to total tau band intensities increased >2-fold by 60 min whereas the AT8 to total tau ratio declined over the same time frame to near zero (Fig. 1D). This trend of persistence of the 12E8 epitope signal was reproducible in a second independent experiment and combined (n = 4), these data reveal a peak increase to 232±69% at 60 min and sustained phosphorylation at 120 min (155±4%, p<0.05) (mean±SEM) (Fig. 1E).

### Redistribution of MAP/tau phosphoepitopes to rod-like or spheroid inclusions in neurites

**Figure 2:**
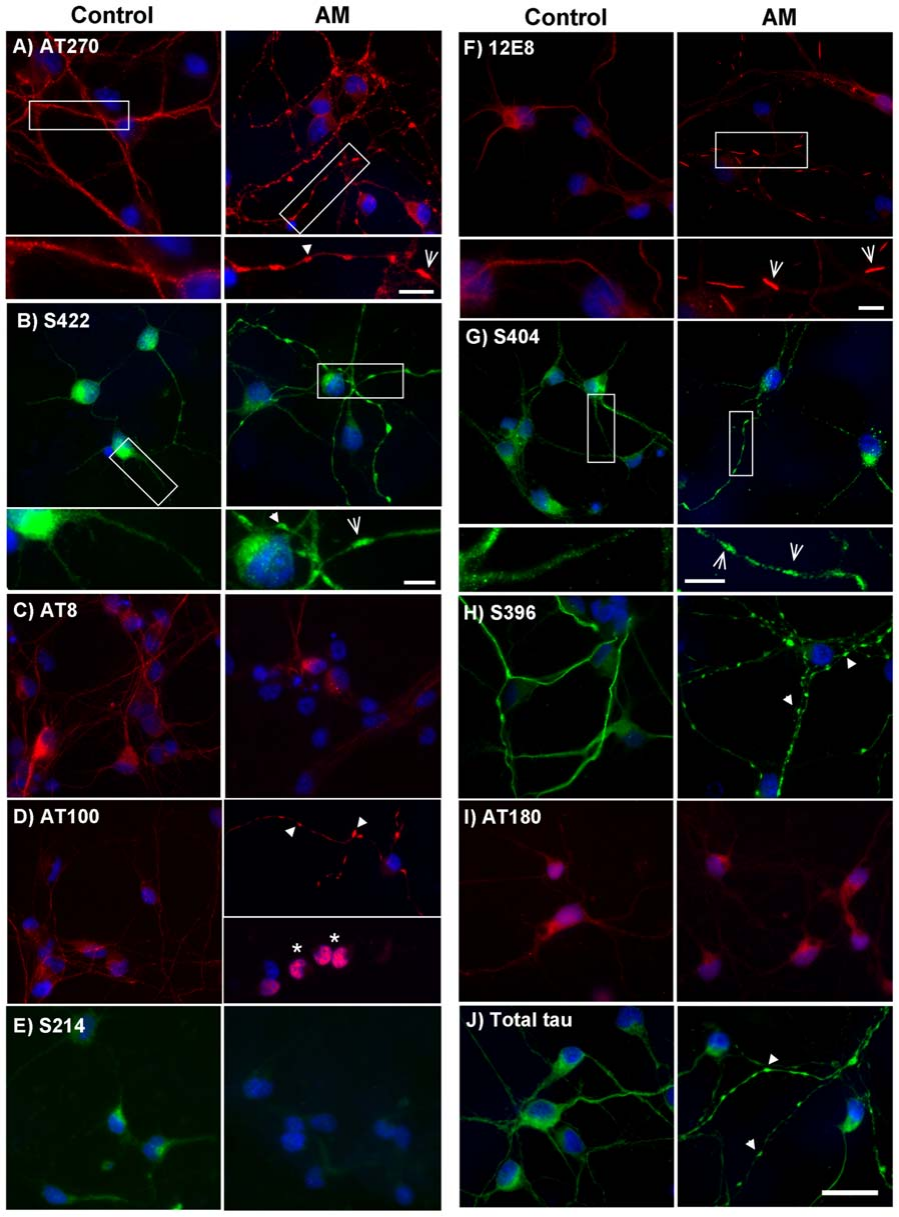
Tau epitopes redistribute in neurons during ATP reduction. Cells were treated with 1 µM AM for 15 min, fixed and permeabilized for 90 sec with 80% methanol or 0.05% Triton-X. Immunostaining was carried out with antibodies against phosphorylated tau epitopes, as indicated. Staining for total tau and all phospho-epitopes was predominantly smooth and evenly distributed in control cells. Following ATP reduction, distribution of AT8 (C), S214 (E) and AT180 (I) epitope labels remained relatively smooth, although staining intensity was reduced. By contrast, AT270 (A), S422 (B), AT100 (D), 12E8 (F), S404 (G) and S396 (H) labeled accumulations that were mostly spheroid in shape, but occasionally rod-like, following ATP-reduction and were frequently observed in tandem arrays along neurites (arrows). Higher magnification insets of controls vs. AM-treated cells are shown for A, B, F, and G. Correspondingly, some spheroid-like accumulation of total tau (J, arrows) was also evident in neurites of AM-treated cells. Interestingly, only aggregates labeled with 12E8 (F) consistently showed classic and distinct rod-shaped structure throughout AM-treated cells, which is better observed at higher magnification (inset in F, arrows). Additionally, ATP reduction led to the redistribution and nuclear-accumulation of AT100 label (D, arrows), a phenomenon that was observed neither in control cultures stained with AT100, nor in treated cells labeled with any other MAP/tau phospho-epitope. However, nuclear AT100 label is unlikely to represent tau (see text). Nuclei were labeled with DAPI (blue). While most epitopes (except for 12E8 – see Fig. 3) appeared identical under methanol and Triton X permeabilization conditions, images in this figure represent a combination of both protocols. Methanol: (A, B, E, G, J). Triton X: (C, D, F, H, I. Scale bar = 20 µm for all images except insets in (A, B, F, and G) = 5 µm.

Next, we conducted immunofluorescence imaging of the same phosphoepitopes in AM-treated (15 min.) neurons to investigate distribution patterns of these antibody labels throughout neurons following mitochondrial inhibition. While some phosphoepitope labeling remained relatively smoothly distributed throughout neurites of AM-treated cells, other labels were aggregated into spheroid inclusions in affected processes (Fig. 2A–J). Cell body staining intensity, indicative of phosphorylation state, of phosphoepitopes AT8, S214 and AT180 was somewhat reduced (AT8 = ∼30% intensity of control cells after 15 min AM treatment), but otherwise comparable to control cells with regards to its smooth distribution. By contrast, AT270, AT100, S396, S404, S422 and total tau labeling aggregated into spheroids that were rarely seen in untreated control cells. Overall cell body staining intensity for some of these epitopes was also found to be reduced (AT270 ∼40% intensity compared to control cells). Although the 12E8 antibody labeled some spheroids, it predominantly labeled rod-shaped aggregates throughout neurites (Fig. 2F) which were previously shown to be AC rods [20]. The insets in Fig. 2 illustrate the appearance of spheroids and rods respectively. Rod-shaped structures were observed less often with the S404, S422 and AT270 phosphoepitope labels (Fig. 2A and G, insets). As shown for AT270 below, these structures only rarely co-localized with AC which was in contrast to 12E8 label which largely colocalized with AC rods.

In AM-treated cells, in addition to labeling neuritic inclusions that co-localized with total tau immunolabeling, AT100 revealed a redistribution into cell nuclei in a subset of neurons (∼36%), as indicated by colocalization with the nuclear stain DAPI (Fig. 2D, asterisks), which was never observed for any other tau epitope (Supplementary Fig. S1). However, further analysis demonstrated that this nuclear epitope does not represent tau protein as AT100 also labeled nuclei of mouse tau knockout neurons (LSM, ITW and JRB unpublished observations). Moreover, neither S214 (which shares the serine-214 recognition site of AT100) nor total tau labels were ever seen to accumulate in nuclei of AM-treated cells further suggesting this AT100 accumulation to be non-tau related. Interestingly however, the same nuclear localization of the AT100 label was seen in post mortem AD tissue (Supplementary Fig. S1F).

**Figure 3:**
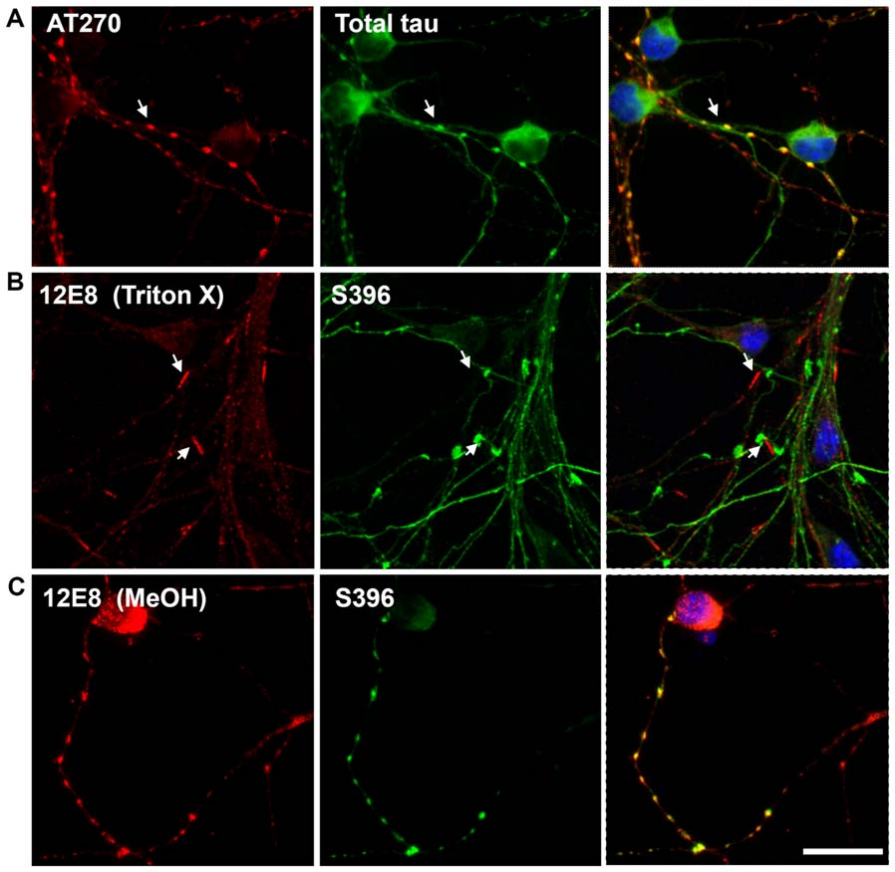
Distinct rod-shaped aggregates are specific to 12E8 and are best preserved with Triton-X permeabilization. ATP-depleted cells were permeabilized for 90 sec with 80% methanol or 0.05% Triton-X and co-labeled for epitopes of tau. (A) Under both methanol and Triton-X methods, aggregations containing AT270 epitopes co-labeled with total tau (shown here with methanol permeabilized cells) suggesting spheroid neuritic aggregates are comprised of tau phosphorylated at numerous epitopes. (B) By contrast, 12E8-labeling was predominantly in distinct rod-shaped aggregates that did not co-label with other phospho-tau epitopes (shown here for S396). 12E8-positive AC rods were best visualized following Triton-X permeabilization. (C) Using methanol permeabilization protocols, 12E8 by contrast more often labeled spheroid structures that as described above could be co-labeled with many other tau epitopes (shown here for S396). Scale bar = 20 µm.

**Figure 4:**
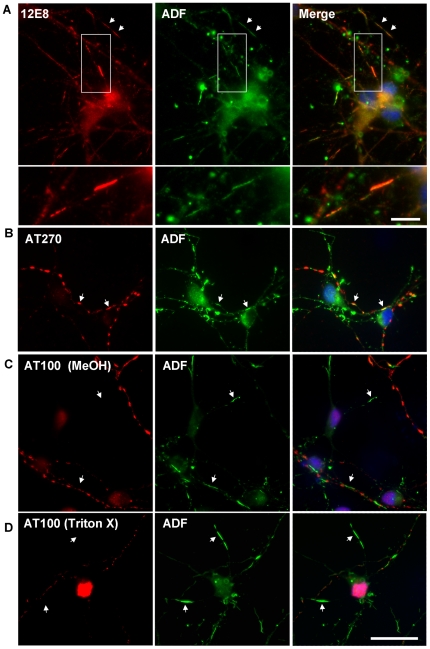
Recruitment to AC rods during ATP reduction is specific for ‘KXGS’ phosphorylated species. (A) Confirmation of 12E8-positive MAP/tau in AC rods during ATP reduction [20]. These inclusions often form in tandem arrays along neurites (arrows). Inset shown at higher magnification in lower panel of (A). Additional double-labeling was performed with AT270 (B) or AT100 (C) and ADF, to see if there was any relationship between these tau epitopes and AC rods. AC rods only sometimes overlapped with AT270 (B, arrows) and were never observed to co-localize with AT100, under either Triton-X or methanol permeabilization (C and D, arrows). These results suggest AC rods are distinct from MAP/tau-containing neuritic spheroids and recruit only the 12E8-phosphorylated MAP/tau isoform into distinct rod structures during ATP reduction. (A, B and D) = Triton X permeabilized. Scale bar = 20 µm; 5 µm (inset in (A)).

Co-labeling studies of spheroids in AM-treated cells revealed co-localization of phospho-tau epitopes with total tau (Fig. 3A and results not shown), suggesting that these observed aggregates in ATP-depleted cells are comprised of tau phosphorylated at many sites. By contrast, rod-like structures immunolabeled for the 12E8 epitope did not co-label with other phosphoepitopes (Fig. 3B). We had previously demonstrated 12E8 label in AC rods [20], which we confirm here in both Triton X and methanol permeabilized cells (Fig. 4A and data not shown). We found that the 12E8 labeling of AC rods in AM treated cells was best preserved under Triton X permeabilization (Fig. 3B) whereas methanol permeabilization resulted mostly in spheroid labeling (Fig. 3C). Further, 12E8-labeled AC rods did not co-localize with S396 label (Fig. 3B) which by contrast only localized to spheroid structures in the same cells (arrows Fig. 3B). This further documents the distinct and separate nature of AC rods versus spheroids.

### 12E8 but not other phosphoepitopes redistributes to AC rods during mitochondrial inhibition

To further investigate whether other tau epitopes are present in AC rods, we carried out double-labeling for ADF and a variety of tau epitopes (since the ADF antibody was rabbit polyclonal, we were restricted to mouse monoclonal MAP/tau antibodies for double-labeling studies). AM-treated cells (15 min) were double-labeled for ADF and AT270 (Fig. 4B), and ADF and AT100 (Fig. 4C, D). AT270 aggregates only occasionally co-localized with AC rods (Fig. 4B, arrows), whereas AT100 label was never seen to co-localize with AC rods after either methanol or Triton-X permeabilization (Fig. 4C, D; arrows). By contrast, as previously shown, 12E8 frequently co-localized with AC rods (Fig. 4A and reference [20]), suggesting this epitope to be of prominent importance in the immediate stages of MAP recruitment to AC rods.

**Figure 5:**
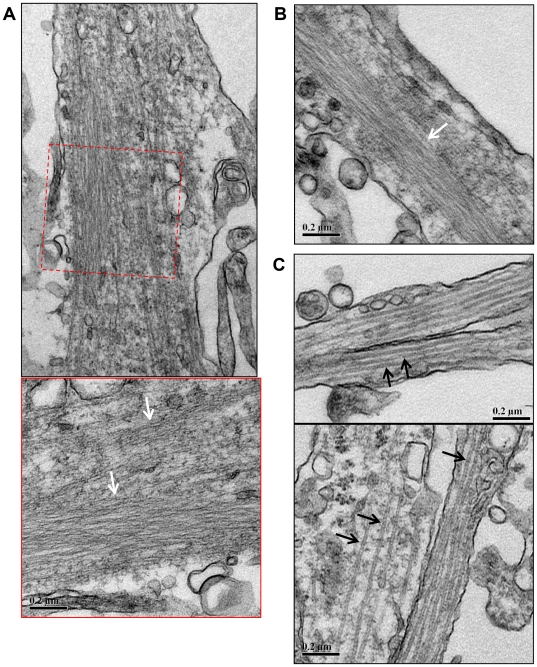
Rod-like accumulations in neurites of primary chick neurons contain densely packed filaments. Transmission electron micrographs of primary chick neurons treated with 1 µM AM for 15 min, fixed and processed for TEM as described in Methods. (A) Densely packed linear arrays of filaments occur in the neurite (white arrows). The lower panel shows a higher magnification view of the boxed region above. (B) Another example of densely packed filaments within a neurite (white arrow). Here the filament bundle spans a large proportion of the width of the neurite. (C) In contrast, unaffected neurites contain individual separated microtubules approximately 24 nm in diameter (black arrows). Scale bars = 0.2 µm.

Ultrastructural EM analysis of AC rods formed in chick neurons following ATP-reduction (Fig. 5A, B) corroborated previous results that demonstrated that AC rods are comprised of many individual parallel filaments arranged into tightly bundled structures [21], [41]–[43]. By contrast, unaffected neurites of treated cells contain individually spaced microtubules approximately 24 nm in diameter (Fig. 5C).

### Actin depolymerization mimics effects of mitochondrial inhibition on 12E8 epitope redistribution but does not affect the distribution of other phosphoepitopes

**Figure 6:**
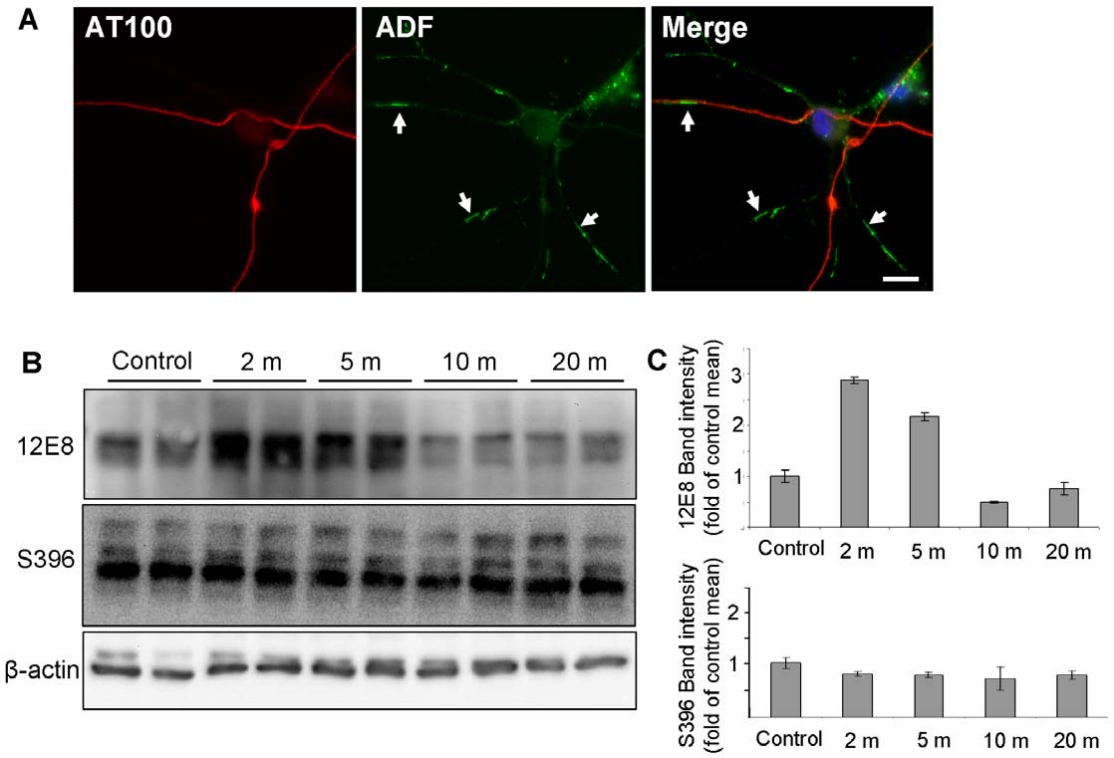
12E8 but not other tau epitopes aggregates and co-localizes with AC rods during actin depolymerization. Primary chick neurons were treated with 1 µg/ml Latrunculin B (Lat B) for 15 min, fixed and immunostained. We first confirmed formation of both AC and 12E8-MAP/tau positive rods in Lat B treated cultures (data not shown), as has previously been observed [20] and then co-stained cultures for ADF with numerous tau epitopes. Whereas an abundance of AC rods were induced by Lat B (A, arrows), label for all tau epitopes, except 12E8, remained relatively smooth and evenly distributed throughout neurites, as shown here for AT100 (A) (other epitopes not shown). Western blots of lysates of Lat B-treated neurons were probed with the same battery of antibodies to tau phospho-epitopes. While most epitopes such as S396 (shown here) showed little change in phosphorylation during Lat B treatment, the 12E8 epitope was strongly phosphorylated within 2 min of treatment (B, C). Band intensities were quantified for each time-point for 12E8 and S396 immunoblots (C) with each lane normalized to individual β-actin loading controls and then calculated as a percentage of control band intensities. At 2 min, 12E8 phosphorylation was 2.9±0.1-fold higher than control means (mean ±min/max intensities). Scale bar = 10 µm.

Similar to mitochondrial inhibition, we previously demonstrated that pharmacologically-induced depolymerization of actin induces the formation of AC rods which subsequently recruit 12E8 epitopes [20]. In order to determine whether actin depolymerization induces redistribution of other phosphoepitopes, we treated primary neurons with Latrunculin B (Lat B) which induces depolymerization of actin filaments in the absence of ATP reduction. The formation and co-localization of AC rods with 12E8 label under these conditions was confirmed (not shown). In contrast to AM-treated cells, staining for other phosphoepitopes including AT100 (Fig. 6A) and S396 (Supplementary Fig. S1D) in Lat B treated cells revealed an even distribution along neurites. Western blot analysis of Lat B-treated neurons revealed a 2.9-fold increase in 12E8 immunoreactivity after 2 min treatment while other phosphoepitopes such as S396 remained largely unchanged (Fig. 6B, C). These results confirm that 12E8 is the primary epitope recruited to Lat B-induced AC rods during actin reorganization, as was also found to be the case for AC rods induced by ATP-reduction. Together these results provide evidence for an important role of KXGS phosphorylation in the initial recruitment of tau or other MAPs to AC rods during times of cellular stress.

## Discussion

Disrupted energy metabolism, mitochondrial dysfunction and increases in oxidative stress in the ageing brain have been linked with the onset of AD pathologies, including aberrant distribution and accumulation of hyperphosphorylated tau and deposition of amyloid-β [24], [44]–[47]. The aim of this study was to investigate whether induced mitochondrial dysfunction could elicit changes in tau phosphorylation and distribution in healthy neurons in cell culture, since mechanisms leading to hyperphosphorylation and redistribution of tau remain elusive, yet are central to understanding the pathogenesis of AD.

Here, we treated primary neuronal cell cultures derived from chick tecta with the mitochondrial inhibitor AM that rapidly depletes cellular ATP [20] and subsequently analyzed changes to phosphorylation and sub-cellular distribution of tau using Western blotting and immunolabeling. Whereas dephosphorylation was observed for every other epitope over 120 min, we observed a substantial increase in phosphorylation at the 12E8 site, an ‘early AD marker’ antibody raised against ‘KXGS’ motifs in the MTBD of human tau [1], [14] (Fig. 1). Notably, we previously demonstrated that this epitope is incorporated into AC rods, which are also generated under ATP depleting conditions [20] (confirmed here in Fig. 4A). The MAP protein family, including the vertebrate proteins tau, MAP2, doublecortin and MAP4 (the latter of which is non-neuronal) contain homologous conserved ‘KXGS’ repeat motifs in MTBDs [8], [48], [49]. In light of this, although tau is probably the most predominant protein recognized by 12E8, we cannot completely discount that the 12E8-immunoreactivity we are observing in AC rods might be due to the presence of other MAPs in addition to tau.

In animal models of ischemia-reperfusion, rapid dephosphorylation of tau occurs during the initial ischemia and we have confirmed in our primary neuronal cell culture model that tau dephosphorylation not hyperphosphorylation is the immediate cellular response to ischemia (mimicked by transient ATP depletion) [50]. An interesting exception to this, consistent in both cell culture and in an animal model, is the enduring phosphorylation of the 12E8 epitope that has been reported in a gerbil-model of brain ischemia [51]. However after reperfusion, tau proteins are slowly phosphorylated and accumulate, resulting in hyperphosphorylation days after the initial ischemic event in the animal potentially due to the down-regulation of phosphatases or induction of specific kinases, although the mechanism remains to be determined [50]. Importantly, our cell culture model is consistent with the animal models and mimics early signaling events directed to tau during transient brain ischemia. While we are not saying that such a large scale global ischemic event is directly relevant to AD, it is interesting that stroke is a significant risk factor for the later development of dementia and AD [50]. More commonly in AD, smaller less severe microischemic events and highly localized cytopathology could reflect a sub-optimal or failing local microvasculature [52].

In contrast to 12E8, other phosphoepitopes including AT270, S422, AT100, S396 and S404 mostly accumulated in small neuritic spheroid swellings in ATP depleted neurons. We found these phosphoepitopes only occasionally co-localized with ADF immunostaining in rod-shaped structures (Fig. 2 and 3), and this observation was rare compared to the high level of co-localization of ADF and 12E8 (Fig. 4). We can conclude that recruitment to AC rods during these conditions is primarily specific to KXGS-phosphorylated species in the initial stages. The occasional rod-shaped structure formed by S404 and AT270 label (Fig. 2) and rare co-localization of AT270 with ADF in AC rods, suggests that other phosphoepitopes of tau may appear in AC rods later, a subject of ongoing investigation. In primary neurons, Lat B-induced actin depolymerization also resulted in specific recruitment of 12E8 epitopes to AC rods, with a concomitant ∼3-fold increase in the signal of the band recognized by 12E8 on immunoblots following 2 min Lat-B treatment (Fig. 6). By contrast, S396 was neither seen to redistribute into spheroid or rod-like aggregates nor deviate from control levels of phosphorylation. These data suggest that actin rearrangement in this cell model triggered either by changes in cellular ATP levels or directly by actin depolymerizing drugs may be an upstream effector of KXGS phosphorylation and redistribution of MAP into AC rods. Converging or independent signaling mechanisms triggered by mitochondrial dysfunction, excess glutamate, oxidative stress, calcium deregulation or other effectors leading to AC rods and MAP/tau redistribution *in vivo* are potentially multifold [22], [43], [47], [53]. Oxidative stress and calcium have previously been implicated in the cytoskeletal pathology of AD and studies have shown both calcium-dependent and –independent pathways leading to AC rods in various model systems [22], [43], [53]. More work is needed to delineate the involvement of these pathways in AD.

A predominant function of tau protein is to bind and stabilize axonal MTs – the tracks for cargo transport to synaptic terminals. Hyperphosphorylation of tau in the MTBD by microtubule affinity regulating kinase (MARK)/PAR1 or other kinases gives rise to a destabilized MT network and concomitant self-assembly of the increased concentration of unbound tau [13], [15], [16]. This self-assembled tau is purported to serve as a seed for further assembly of redistributed tau into PHFs along neurites (neuropil threads) and in the somatodendritic domain (neurofibrillary tangles) [1], [2], [54], [55]. Albeit in a cell model and not demonstrated *in situ* in brain tissue, our results suggest that AC rods may contribute to the sequestration and accumulation of unbound tau via its phosphorylated MTBD in neurites leading to neuropil thread-like inclusions and potentially PHFs. We speculate that rapid depolymerization and rearrangement of the actin cytoskeleton during rod assembly may induce activation of MARK/Par-1 or other kinases, the former of which has previously been implicated in conferring tau toxicity in Drosophila models [15], [56], [57]. Consistent with this, activation of MARK correlates with elevated phosphorylation and dendritic accumulation of tau in stressed primary hippocampal neurons [47].

Extensive studies have demonstrated a role for tau and other MAPs in cross-linking and bundling of actin filaments [28]–[37]. Some studies have additionally demonstrated that MAP-actin association is regulated by the reversible phosphorylation of MAPs and that furthermore, tau and MAP2 interactions with actin occur via KXGS-containing motifs in the repeat regions of their MTBD domains [31], [32], [58]. That 12E8 labeled epitopes co-localized with AC rods in our study further supports a central role for KXGS motifs in the rapid association with actin. Moreover, EM analysis of AC rods following 15 min AM treatment in primary neurons demonstrate the presence of filamentous bundles (Fig. 5), and we speculate that AC-actin bundles form initially and serve as nucleation sites for subsequent recruitment of 12E8 epitopes. A recent study of human AD brains provides some support for this notion, which reported a correlation between increased cofilin expression and tau pathology in specific regions of the brain, including the temporal cortex [59]–[61]. Expression of the microRNA miR-107 has been shown to decrease in AD brain [60], which is associated with increased amyloid precursor protein and cofilin protein levels and formation of cofilin rod-like structures [61]. In regions of AD brains with confirmed reduction of miR-107 expression, a significant increase in NFTs was observed when compared with adjacent tissue expressing normal levels of miR-107 [59].

Further investigations are required to delineate the mechanisms facilitating the interaction between MAP and AC-actin rods and whether it is direct or indirect. Important questions that remain to be answered are whether the KXGS phosphorylation of tau or other MAPs is causative or correlative with its association with actin or AC rods and what mechanism facilitates the interaction between 12E8 epitopes and AC rods. Recent characterization of isolated AC rods demonstrated the presence of the scaffolding protein 14-3-3ζ [42] which was also shown to be associated with 12E8 within AC rods (unpublished observations LSM and JRB), suggesting that 14-3-3ζ may have a role either in rod structure or in a longer time-dependent maturation of MAP/tau phosphorylation, as has previously been suggested in other studies [62]–[64]. Answering these questions is the subject of ongoing studies.

In summary, we have shown that mitochondrial inhibition is associated with specific and sustained detection of 12E8 epitopes indicative of MAP phosphorylation in MTBD KXGS motifs, but a concomitant dephosphorylation of other phospho-tau epitopes. The combined effects of MT destabilization and accumulation of MAP/tau and cofilin-actin rods in the neurites is proposed to inhibit axonal transport, ultimately leading to synaptic pruning and neuronal death [47], [65]–[68]. The present findings therefore suggest that targeting disrupted energy metabolism, mitochondrial dysfunction and downstream actin reorganization in aging cells may help prevent neurodegeneration associated with aberrant phosphorylation, redistribution and self-assembly of tau.

## Supporting Information

Figure S1 AT100 labels a non-tau component in the nucleus during mitochondrial inhibition, actin depolymerization and in postmortem AD brain. Primary chick neurons were treated with 1 µM AM or 1 µg/ml Lat B for 15 min, fixed, immunostained and analyzed via confocal laser scanning. ATP reduction with AM induced nuclear accumulation of the AT100 label in a proportion of neurons (36±2%; mean±SEM), as indicated by co-localization with DAPI (A, B). Co-stainings revealed no obvious translocation of total tau into the nuclear compartment in cells exhibiting AT100 nuclear accumulation (B, arrows), suggesting the AT100 antibody is labeling a protein other than tau. AT100 labeling in untreated cells (C) remained contained in the neurites. Similarly, actin depolymerization with Lat B gave rise to nuclear accumulation of AT100 label in 22±2% cells (mean±SEM) (D) and again, lack of total tau and other epitopes such as S396 (D) in the nucleus. Interestingly, pre-treating cultures for 2 min with the F-actin stabilizing drug jasplakinolide prevented nuclear accumulation of AT100 in cultures subsequently treated with either AM or Lat-B (data not shown). Formalin-fixed 7 µm brain sections from the hippocampal region of confirmed AD cases were immunostained for phospho-tau epitopes. S396 label (E) and other tau epitopes (data not shown) often accumulated in flame-shaped tangles, though remained largely absent from the nucleus (E, arrows). By contrast, double-labeling studies showed AT100 label frequently appearing in the nuclear compartment as evidenced by its co-localization with DAPI (F, arrowhead). In the same neurons, S396 was exclusively contained within the somal compartment (F, arrow). Although characterization and identification of this AT100-positive protein is beyond the scope of the present study, further investigation of this stress-induced response would be valuable as the appearance of the epitope in the nucleus specifically occurs in stressed cells (ATP depleted) or AD brain and not control cells. Images in (A–D): confocal microscope; (E, F): optical microscope. Scale bars = 10 µm (A, F); 20 µm (B–E).
(TIF)
