# Supplementary figures and images for: Rapid Changes in Phospho-MAP/Tau Epitopes during Neuronal Stress: Cofilin-Actin Rods Primarily Recruit Microtubule Binding Domain Epitopes
Source: PLoS One. 2011 Jun 28;6(6):e20878. doi: 10.1371/journal.pone.0020878 (PMC3125162; doi:10.1371/journal.pone.0020878)

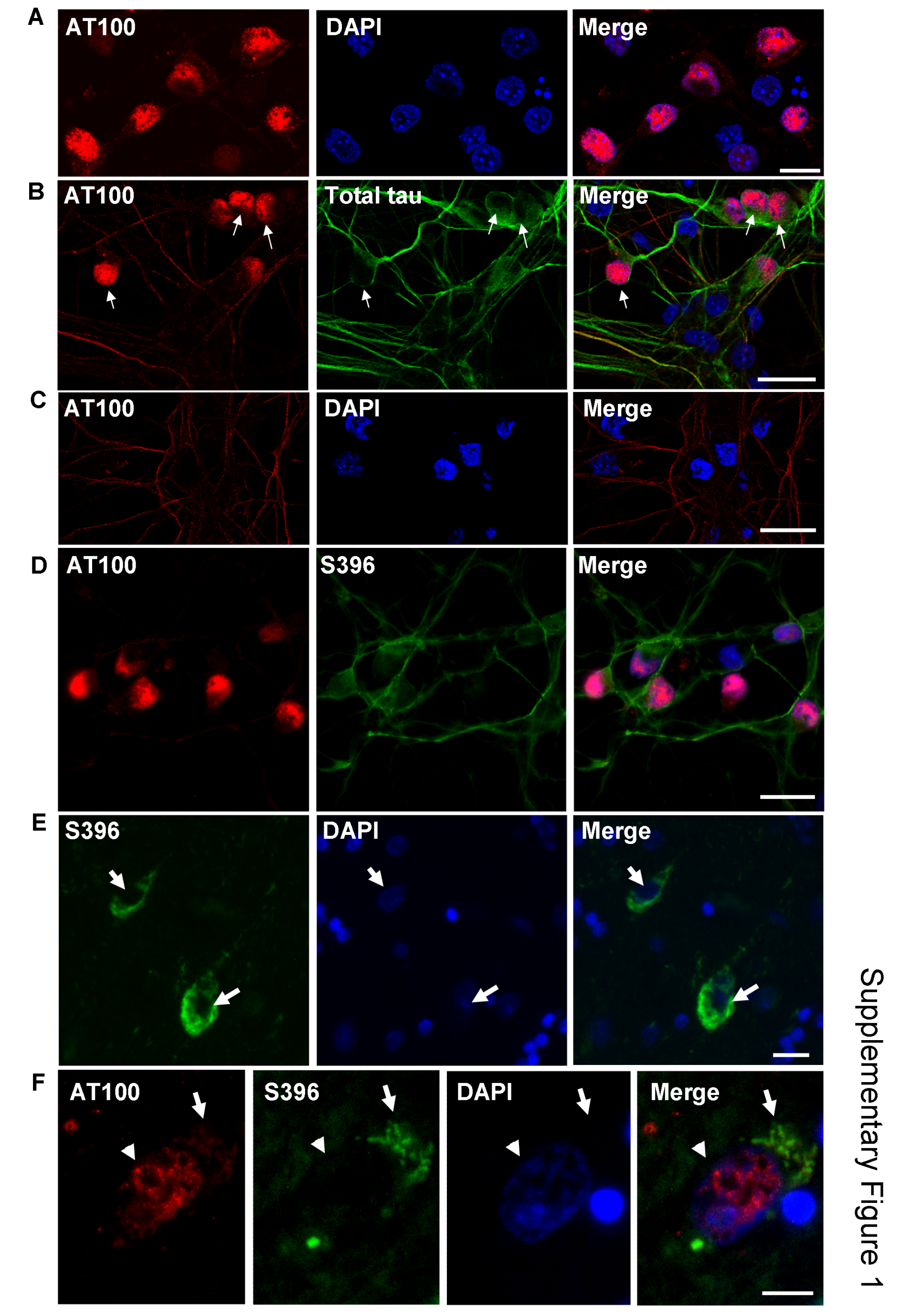

Supplement: Figure S1 — AT100 labels a non-tau component in the nucleus during mitochondrial inhibition, actin depolymerization and in postmortem AD brain. Primary chick neurons were treated with 1 µM AM or 1 µg/ml Lat B for 15 min, fixed, immunostained and analyzed via confocal laser scanning. ATP reduction with AM induced nuclear accumulation of the AT100 label in a proportion of neurons (36±2%; mean±SEM), as indicated by co-localization with DAPI (A, B). Co-stainings revealed no obvious translocation of total tau into the nuclear compartment in cells exhibiting AT100 nuclear accumulation (B, arrows), suggesting the AT100 antibody is labeling a protein other than tau. AT100 labeling in untreated cells (C) remained contained in the neurites. Similarly, actin depolymerization with Lat B gave rise to nuclear accumulation of AT100 label in 22±2% cells (mean±SEM) (D) and again, lack of total tau and other epitopes such as S396 (D) in the nucleus. Interestingly, pre-treating cultures for 2 min with the F-actin stabilizing drug jasplakinolide prevented nuclear accumulation of AT100 in cultures subsequently treated with either AM or Lat-B (data not shown). Formalin-fixed 7 µm brain sections from the hippocampal region of confirmed AD cases were immunostained for phospho-tau epitopes. S396 label (E) and other tau epitopes (data not shown) often accumulated in flame-shaped tangles, though remained largely absent from the nucleus (E, arrows). By contrast, double-labeling studies showed AT100 label frequently appearing in the nuclear compartment as evidenced by its co-localization with DAPI (F, arrowhead). In the same neurons, S396 was exclusively contained within the somal compartment (F, arrow). Although characterization and identification of this AT100-positive protein is beyond the scope of the present study, further investigation of this stress-induced response would be valuable as the appearance of the epitope in the nucleus specifically occurs in stressed cells (ATP depleted) or AD brain and [file pone.0020878.s001.tif]
